# Supplementary material for: Exploring the Role of a Putative Secondary Metabolite Biosynthesis Pathway in Mycobacterium abscessus Pathogenesis Using a Xenopus laevis Tadpole Model
Source: Microorganisms. 2024 May 31;12(6):1120. doi: 10.3390/microorganisms12061120 (PMC11206028; doi:10.3390/microorganisms12061120)
Supplement: Supplementary file 1 [file microorganisms-12-01120-s001.zip › microorganisms-3006919-supplementary.pdf]

Table S1: List of qPCR primer sequences.

**PRIMER**	**SEQUENCE (5'-3')** [a]
GAPDH	F: GACATCAAGGCCGCCATTAAGACT<br>R: AGATGGAGGAGTGAGTGTCACCAT
TNF-α	F: TGTCAGGCAGGAAAGAAGCA<br>R: CAGCAGAGCAAAGAGGATGGT
IL-1β	F: CATTCCCATGGAGGGCTACA<br>R: TGACTGCCACTGAGCAGCAT
Type I IFN	F: GCTGCTCCTGCTCAGTCTCA<br>R: GAAAGCCTTCAGGATCTGTGTGT
iNOS	F: GCTGCTCCTGCTCAGTCTCA<br>R: GAAAGCCTTCAGGATCTGTGTGT

\

[a] F: Forward; R:Reverse
